# Association between Obesity and Omega-3 Status in Healthy Young Women

**DOI:** 10.3390/nu12051480

**Published:** 2020-05-20

**Authors:** Isabel E. Young, Helen M. Parker, Rebecca L. Cook, Nicholas J. O’Dwyer, Manohar L. Garg, Kate S. Steinbeck, Hoi Lun Cheng, Cheyne Donges, Janet L. Franklin, Helen T. O’Connor

**Affiliations:** 1 Faculty of Medicine and Health, School of Health Sciences, The University of Sydney, Sydney, NSW 2006, Australia; h.parker@sydney.edu.au (H.M.P.); rebecca.cook@live.com.au (R.L.C.); nicholas.odwyer@sydney.edu.au (N.J.O.); helen.oconnor@sydney.edu.au (H.T.O.); 2 Charles Perkins Centre, The University of Sydney, Camperdown, NSW 2006, Australia; kate.steinbeck@health.nsw.gov.au (K.S.S.); helen.cheng@health.nsw.gov.au (H.L.C.); 3 School of Biomedical Sciences and Pharmacy, University of Newcastle, Callaghan, NSW 2308, Australia; manohar.garg@newcastle.edu.au; 4 Faculty of Medicine and Health, Discipline of Child and Adolescent Health, The University of Sydney Children’s Hospital Westmead, Westmead, NSW 2145, Australia; 5 School of Exercise Science, Sport and Health, Charles Sturt University, Bathurst, NSW 2795, Australia; cheyne_donges@outlook.com; 6 Metabolism and Obesity Services, Royal Prince Alfred Hospital, Camperdown, NSW 2050, Australia; janet.franklin@sswahs.nsw.gov.au

**Keywords:** obesity, omega-3, n-3 PUFA, omega-3 index, young women

## Abstract

Omega-3 polyunsaturated fatty acids (n-3 PUFAs) are essential for healthy development and protect against metabolic disease. However, individuals with obesity may be pre-disposed to experiencing lower n-3 PUFA status than normal-weight individuals. This cross-sectional study examined the relationship between the omega-3 index (O3I), body mass index (BMI) and dietary intake in healthy young women (n = 300; age = 18–35 y), a group not previously focused on. Intake was adjusted for energy using the residuals method, and associations were explored using independent *t*-tests and Pearson’s correlations. Participants with obesity were found to have significantly lower O3I than normal-weight participants (*p* < 0.0001); however, no significant differences were observed in mean n-3 PUFA intakes. Even so, energy-adjusted intakes of n-3 PUFAs, with the exception of alpha-linolenic acid, were significantly correlated with O3I. This study demonstrates that O3I is influenced by both BMI and diet in young women; however the relationship between these two variables may be complex. Current intakes of n-3 PUFA observed in young women may not be effective in achieving target O3I levels in those with obesity, and further research is needed to find effective ways of improving n-3 PUFA status in a group already at increased risk of metabolic disease.

## 1. Introduction

Rates of obesity in Australia are increasing, with 28% of adults currently classified as obese, and a total of 67% of adults classified as overweight or obese [1]. Obesity is characterised by excessive accumulation of adipose tissue and is associated with higher risk of metabolic diseases and other comorbidities [2]. Obesity has also been linked with excess energy intake and poor diet quality, with intake of discretionary foods, saturated and trans fats being linked with higher body mass index (BMI) [3]. Omega-3 polyunsaturated fatty acids (n-3 PUFAs) have emerged as a potential protective nutrient against the cardiometabolic risks associated with obesity [4], but higher BMI has also been linked with low omega-3 status among adults [5,6,7].

In humans, the synthesis of n-3 PUFAs cannot occur endogenously and there is poor conversion from parent fatty acids [8,9,10]. Therefore, n-3 PUFAs, including alpha-linolenic acid (ALA), eicosapentaenoic acid (EPA), docosapentaenoic acid (DPA) and docosahexaenoic acid (DHA), are an important component of the human diet [8,9]. n-3 PUFAs play key roles in growth and development, and also the maintenance of optimal health across all age groups [4,11,12]. The composition of fatty acid intake has been shown to influence the composition of phospholipids in the cell membrane, with a higher intake of n-3 PUFA leading to a greater proportion of n-3 PUFA in the cell membrane [13,14]. This accumulation of n-3 PUFA is protective against metabolic diseases, can alter cell membrane function and metabolism, and decreases circulatory inflammatory markers [14,15,16,17,18,19]. Previous research has also shown that a higher intake of n-3 PUFA may be beneficial in management of obesity, which is considered a chronic state of inflammation [20,21]. In contrast, omega-6 polyunsaturated fatty acids (n-6 PUFAs) are pro-inflammatory [22], with higher intakes being linked to increased incidence of obesity and metabolic diseases [23].

Despite the importance of dietary n-3 PUFAs, young women typically consume less than half the amount that is expected to be protective against chronic disease [24,25,26], and only 10% of women of child-bearing age consume the recommended intake of DHA [27]. In addition to low n-3 PUFA intake, young women also gain weight at a more rapid rate than their older counterparts [28,29], making them a particularly vulnerable group for low n-3 PUFA status. Young women are also susceptible to metabolic diseases such as polycystic ovarian syndrome (PCOS) with insulin resistance and hyperandrogenism, potentially increasing vulnerability to low n-3 PUFA status [30,31]. However, in contrast, some young women also take hormonal birth control, which can alter n-3 PUFA metabolism, potentially protecting them from low omega-3 status [31]. There is also an inherent difference between the sexes in the n-3 PUFA content of tissues [32,33], highlighting the need for focussed studies on young women.

The measurement of n-3 PUFA status is not typically undertaken as part of routine blood tests, but it can provide important information relevant to an individual’s health. The omega-3 index (O3I), which is the proportion of EPA and DHA in erythrocyte cell membrane lipids, can assess medium-term body status (~3 months) of n-3 PUFAs and corresponds with body tissue levels of n-3 PUFA [14,34,35,36]. Previous work in other population groups found that a low O3I is associated with a higher BMI, waist circumference and weight [5,37], but this has not yet been examined in young women.

This study is a secondary analysis of the cross-sectional “Food Mood and Mind” study and aims to explore the association between O3I and BMI in healthy young women.

## 2. Materials and Methods

### 2.1. Study Design and Participants

The Food Mood and Mind study [38,39] was a cross-sectional study that recruited a convenience sample (target n = 300) of young (18–35 y) healthy weight (HW) (n = 150; BMI: 18.5–24.9 kg/m^2^) and obese weight (OB) (n = 150; BMI: ≥30 kg/m^2^) women. Participants were recruited from both urban and rural areas of New South Wales, with one third recruited from a major rural centre (Bathurst; n = 100; 50% HW, 50% OB). The study was approved by the Human Research Ethics Committees linked to local health district services and participating universities (protocol numbers: 2014/050, X10-0259 and HREC/10/RPAH/455).

### 2.2. Inclusion and Exclusion Criteria

Participants who were healthy and had a BMI within either the normal or obese weight category according to the WHO guidelines (BMI 18.5–24.9 and ≥30 kg/m^2^, respectively) [40] were eligible. Participants were excluded if they had significant medical conditions (cardiovascular, renal or metabolic disease including type 2 diabetes, malignancies), were pregnant or breastfeeding, currently using medications or substances that are known to alter mood, or inadequate facility in spoken and written English. Participants were screened for eligibility via telephone using a standardised proforma and written informed consent was obtained prior to participation in the study.

Participants with incomplete data sets (n = 23), those who were extreme energy reporters (<3280 kJ, >19,500 kJ per day; n = 12) [41,42] or those whose C-reactive protein exceeded 10 mg/L, indicating the possibility of an underlying disease or infection not known to the participant (n = 20) [43], were excluded in a stepwise manner (n = 55).

### 2.3. Data Collection

The data collected included anthropometric measures, a validated food frequency questionnaire (FFQ) and a fasting blood sample.

#### 2.3.1. Anthropometry

Participants wore light clothing and no shoes for anthropometric data collection. Weight was recorded on an electronic digital platform scale (PW-200KGL, Thebarton, Adelaide, Australia) to the nearest 0.1 kg. Height was recorded in duplicate with a stadiometer (213 portable stadiometer; SECA, Hamburg, Germany) to the nearest 0.1 cm. Waist circumference was recorded in duplicate to the nearest 0.1 cm at the mid-point between the lowest rib and the iliac crest using a retractable metal tape (Lufkin W606PM; Cooper Industries, Sparks, NV, USA) according to the International Diabetes Federation Guidelines [44]. Instruments used for measurements were calibrated and methods validated internally (height and weight) or externally (waist circumference).

#### 2.3.2. Blood Collection and Biochemical Analysis

A fasting (12 h) blood draw was collected, from which O3I was analysed in a university laboratory via trans-esterification of the washed erythrocyte fraction of blood, and gas chromatography using a fixed carbon-silica column 30 m × 0.25 mm (DB-225) (J and W Scientific) [45]. The O3I was used as an indicator of overall body n-3 PUFA status and was calculated as the percentage of EPA plus DHA as a proportion of total erythrocyte membrane fatty acids [35,45,46].

#### 2.3.3. Food Frequency Questionnaire (FFQ)

Habitual dietary intake was measured using a semi-quantitative FFQ (the Dietary Questionnaire for Epidemiological Studies Version 2) [47], which has been validated for use in the Australian population [48]. The FFQ includes 74 food items and allows respondents to select an estimate of their usual portion size for the consumption of common foods and beverages (including alcohol). Analysis of the FFQ was automated and performed by the Cancer Council of the state of Victoria, with results provided as average daily intake of each food/beverage item (grams) and daily total energy and nutrient intakes.

Intake of each of the food groups was determined using the Australian Guide to Healthy Eating (AGHE) [49]. Individual food items were grouped according to the AGHE and their total weight divided by their standard serve size (grams) to provide total serves per day. Nutrient and food group data were adjusted for total energy intake using the residuals model [50].

#### 2.3.4. Additional Data Collection

Participant characteristics collected included ethnicity, years of education, level of completed education, use of the oral contraceptive pill (OCP) and geographic location (urban/rural). The International Physical Activity Question (IPAQ)—Short Form, an internationally validated tool, was used to estimate physical activity in Metabolic Equivalent of Task (MET)—minutes per week [51].

### 2.4. Statistical Analyses

Data were collated and screened prior to analysis.

Associations between variables were investigated using correlational and multiple regression analyses for the entire cohort and for the HW and OB groups separately. Independent samples t-tests were used to compare participant characteristics between HW and OB groups. The O3I was compared between HW and OB groups using one-way analyses of variance (ANOVAs), extended to analyses of covariance (ANCOVAs) with energy-adjusted intakes for total n-3 PUFA, DHA, EPA, DPA and ALA as covariates. BMI was analysed across quartiles of the O3I and erythrocyte n-3 PUFA via ANOVA with planned trend contrasts. The effect of fish oil supplements was analysed for HW and OB groups via two-way ANOVA. Statistical significance was set at *p* < 0.05. Tukey post hoc tests were used to identify the locus of significant effects detected on ANOVA or ANCOVA. All analyses were completed using Statistica (v.13, TIBCO Software Inc., CA, USA.).

## 3. Results

The cross-sectional study recruited 299 healthy young women (18–35 y), of whom n = 244 with complete data sets for the outcomes of interest were included in all statistical analyses. Participants were classified as either OB or HW based on BMI. Their characteristics are summarised in Table 1.

Participants were aged 18–35 y with the OB group being slightly older on average than the HW group. As anticipated, mean BMI and waist circumference were significantly different between the OB and HW groups. CRP was significantly higher in the OB group. Intakes of EPA, DPA and DHA were not different between the groups; however, OB participants had a significantly higher intake of ALA. Only 40.6% of the entire cohort met suggested dietary targets (SDT) for n-3 PUFAs and there was no significant difference in the proportion of women in the HW and OB groups meeting the SDT [52].

**Figure 1:**
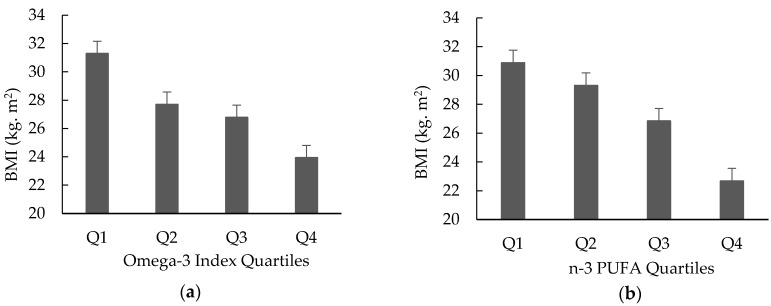
Mean BMI across quartile groups of (**a**) O3I, and (**b**) erythrocyte n-3 PUFA (means ± standard errors).

O3I and n-3 PUFA were significantly different between the OB and HW groups, with the participants with obesity having a lower O3I and overall levels of erythrocyte n-3 PUFA than HW participants. Indeed, a clear linear trend (*p* < 0.0001) was observed of decreasing BMI across increasing quartiles of both O3I and erythrocyte n-3 PUFA (Figure 1a,b). The group differences remained significant (*p* < 0.001) when adjusted (both separately and together) for energy-adjusted intakes of total n-3 PUFA, DHA, EPA, DPA and ALA as covariates, suggesting that the relationship between O3I and obesity is independent of total intake of n-3 fatty acids and individual n-3 PUFAs.

As shown in Table 2, when adjusted for energy intake, intake of EPA, DPA and DHA was significantly positively correlated with n-3 PUFA status in both the HW and OB groups. ALA was not significantly correlated with either O3I or n-3 PUFA. In most cases, the correlation between energy-adjusted intake and biochemistry was stronger in HW participants than OB participants, suggesting that the impact of n-3 PUFA intake may be dampened in individuals with obesity.

The O3I was not found to be significantly affected by use of the oral contraceptive pill (*p* = 0.90; n = 62 using, n = 151 not using). However, although reported by only a few participants (n = 11), taking fish oil supplements resulted in a significantly higher mean O3I across the entire cohort (*p* = 0.024). Post hoc tests showed that within the HW group, those taking fish oil supplements (n = 5) had significantly higher O3I (*p* = 0.002) compared with those not taking supplements (n = 133); whereas in the OB group, there was no significant difference between those taking (n = 6) and those not taking (n = 100) supplements (*p* = 0.99).

## 4. Discussion

In this cross-sectional study, O3I was significantly negatively associated with BMI, waist circumference and CRP in healthy young women (18–35 y). These relationships were also found to be independent of n-3 PUFA intake and, as the participants reported being healthy with no comorbidities, independent of other health concerns. These results suggest that there may be altered metabolic pathways in the absorption, utilisation and/or storage of n-3 PUFA in people with obesity leading to lower omega-3 status.

This relationship between omega-3 status and bodyweight status has been shown previously in other population groups including older adults, children and adolescents. These studies also found a significant negative correlation between increased BMI or weight status and omega-3 status (either plasma n-3 PUFA or O3I) [5,6,7,37,53,54]. Whilst the mechanism for this negative relationship between body dimensions and O3I is not yet fully understood, it has been suggested that there may be altered metabolic pathways in individuals with obesity [55,56,57,58,59], resulting in lower plasma n-3 PUFA or perhaps lower uptake into target body tissues such as erythrocyte membranes [60]. The reverse has also been suggested whereby a higher omega-3 status may be protective against obesity, as n-3 PUFA increases basal fat oxidation, in turn reducing overall fat mass and BMI [56]. Indeed, a high intake of n-3 PUFA has previously been shown to be negatively associated with central adiposity and presence of obesity [61]. Proposed mechanisms for omega-3 as protective against obesity include the anti-inflammatory effect of n-3 PUFA [12], which may lead to prevention of the low-grade chronic inflammation that is often found in conditions of excess adiposity such as obesity and which contributes to raised cardiometabolic risk [62]. n-3 PUFA has also been shown to affect appetite regulation, increasing satiety in overweight and obese individuals during weight loss [63]. However, while it is unknown whether the presence of obesity or low n-3 PUFA status comes first, this decreased omega-3 status in individuals with obesity may further pre-dispose them to metabolic diseases including increased inflammation.

Whilst energy-adjusted intakes of n-3 PUFAs, except ALA, were correlated with both O3I and total erythrocyte n-3 PUFA, the relationship between BMI and omega-3 status remained significant when intake was accounted for as a covariate, suggesting an independent effect of obesity on omega-3 status, over and above dietary intake. Similar results have also been shown in children, where BMI *z*-scores were found to be significantly negatively associated with total n-3 PUFA and DHA despite the children with higher BMI *z*-scores having a higher intake of fatty acids including n-3 PUFA [53]. This suggests that, in individuals with obesity, a higher intake of n-3 PUFA may not result in a significant increase in O3I, or a given intake of n-3 PUFA may have a smaller effect on O3I than in a HW individual. This idea is further supported by the results of the present study where taking fish oil supplements was associated with significantly higher n-3 PUFA status in healthy-weight, but not in obese-weight participants. However, only 11 participants reported taking fish oil supplements, and further research is warranted to test this finding in a larger sample. Whilst it has previously been shown that supplementation can improve n-3 PUFA status in healthy individuals [64], other studies have also found that “priming” the body with supplemental omega-3 prior to a weight loss program can result in enhanced weight loss [21], indicating there may yet be an effect of increasing dietary intake of omega-3 in those with obesity that may not be perceptible via changes to O3I.

In the current study, use of the oral contraceptive pill (OCP) was not found to significantly alter O3I. This contradicts evidence that shows that the progesterone released by the OCP to mimic constant luteal conditions can cause an upregulation of n-6 PUFA conversion to n-3 PUFA [31]. However, in our study, OCP use data were incomplete, and participants were not asked to identify the composition or specific brand of OCP they were using nor the length of time they had been on the OCP, and consistency of use was also not collected.

Strengths of this research include the use of O3I, with previous studies instead using plasma n-3 PUFA or serum phospholipid fatty acids, which are highly influenced by recent diet [14]. The strength of using O3I lies in its being unaffected by previous-day dietary intake and its relative stability over time, and hence it is considered to be indicative of n-3 PUFA status and intake over a three-month period [34,35]. The participants of this study were healthy, decreasing the effect of possible health-related confounders. Women who were currently pregnant, breastfeeding or less than 6 months post-partum were also excluded, eliminating the effect that pregnancy may have on n-3 PUFA status, metabolism and in vivo synthesis [65,66]. Limitations of this study include the self-reported nature of dietary intake, with food frequency questionnaires commonly being associated with both over- and underestimation of dietary intake [67,68,69]. Whilst women were screened for metabolic disease and medical conditions, they were not tested for PCOS, which can often go undiagnosed and may have impacted n-3 PUFA status in the young women [30,31,70]. Furthermore, while we collected anthropometry data including BMI and waist circumference, more in-depth body composition data would have yielded information about adipose tissue volumes. This may contribute to differences observed in erythrocyte omega-3 due to the increased volume of distribution for fatty acids absorbed from the diet, and therefore a lower apparent blood status of omega-3 in those with larger adipose tissue stores.

## 5. Conclusions

This research supports the current literature whereby individuals with overweight or obesity have significantly lower O3I or n-3 PUFA status than their healthy-weight counterparts. In healthy young women, this relationship between BMI and O3I was found to be independent of n-3 PUFA intake. Participants taking fish oil supplements were found to have significantly higher O3I than those who did not, suggesting their importance in maintaining a higher level of n-3 PUFA in cell membranes. However, this effect was primarily observed in healthy-weight individuals, and the effect of omega-3 supplements in participants with obesity was less clear. Further research is necessary to elucidate the mechanisms by which increased BMI and obesity affect n-3 PUFA status, and how this relates to risks of metabolic disease among individuals with obesity.

## Figures and Tables

**Table 1:** Characteristics of OB and HW participants.

Characteristic	Total Group (n = 244)	HW (n = 138)	OB (n = 106)	*p*-Value *
Age (years)	25.7 ± 5.1	24.8 ± 4.6	26.8 ± 5.4	0.002
Location (n, %)				
Urban	165	96	69	0.46
Rural	79	42	37	
Years of education	16.2 ± 2.2	16.6 ± 2.2	15.8 ± 2.1	0.009
BMI (kg/m^2^)	27.4 ± 7.3	21.8 ± 1.7	34.8 ± 4.6	<0.001
Waist circumference (cm)	82.4 ± 16.9	69.5 ± 4.4	99.1 ± 11.5	<0.001
Biochemistry ^1^				
O3I (%)	6.4 ± 1.6	6.7 ± 1.5	5.9 ± 1.5	<0.001
n-3 PUFA (%)	11.2 ± 2.9	12.3 ± 3.0	9.8 ± 2.1	<0.001
ALA (%)	0.31 ± 0.18	0.33 ± 0.18	0.28 ± 0.18	0.070
EPA (%)	0.94 ± 0.40	0.93 ± 0.44	0.96 ± 0.34	0.61
DPA (%)	4.6 ± 2.1	5.3 ± 2.3	3.6 ± 1.2	<0.001
DHA (%)	5.4 ± 1.4	5.8 ± 1.3	4.9 ± 1.4	<0.001
CRP (mg/L)	2.3 ± 2.4	1.2 ± 1.4	3.7 ± 2.6	<0.001
Intake				
ALA (mg/day)	1018 ± 513	958 ± 458	1095 ± 569	0.04
EPA (mg/day)	129 ± 147	130 ± 156	128 ± 135	0.91
DPA (mg/day)	48 ± 41	47 ± 43	48 ± 37	0.78
DHA (mg/day)	278 ± 288	277 ± 305	279 ± 265	0.95

Data reported as mean ± standard deviation. * *p*-value for HW versus OB, significance set at *p* < 0.05; *t*-test for continuous data, chi-square test for categorical data; ^1^ data for erythrocyte fatty acids are presented as proportion of total erythrocyte fatty acids. Abbreviations: OB, obese weight; HW, healthy weight; BMI, body mass index; O3I, omega-3 index; n-3 PUFA, n-3 polyunsaturated fatty acids; ALA, alpha linolenic acid; EPA, eicosapentaenoic acid; DPA, docosapentaenoic acid; DHA, dosocahexaenoic acid; CRP, C-reactive protein.

**Table 2:** Correlations between energy-adjusted n-3 PUFA intake and O3I and n-3 PUFA.

Energy-Adjusted Intake	HW (n = 138)		OB (n = 106)	
	O3I	n-3 PUFA	O3I	n-3 PUFA
ALA (mg/day)	*r* = −0.15	*r* = −0.08	*r* = −0.17	*r* = −0.11
	*p* = 0.086	*p* = 0.345	*p* = 0.085	*p* = 0.282
EPA (mg/day)	*r* = 0.42	*r* = 0.27	*r* = 0.37	*r* = 0.19
	*p* < 0.001	*p* = 0.001	*p* < 0.001	*p* = 0.048
DPA (mg/day)	*r* = 0.44	*r* = 0.27	*r* = 0.44	*r* = 0.24
	*p* < 0.001	*p* = 0.001	*p* < 0.001	*p* = 0.014
DHA (mg/day)	*r* = 0.42	*r* = 0.26	*r* = 0.37	*r* = 0.19
	*p* < 0.001	*p* = 0.002	*p* < 0.001	*p* = 0.049

Data presented: *r*-value (Pearson’s correlation) for relationship between intake of n-3 PUFA and erythrocyte fatty acid composition; significance set at *p* < 0.05. Abbreviations: HW, healthy weight; OB, obese weight; O3I, Omega 3 Index; n-3 PUFA, n-3 polyunsaturated fatty acids; ALA, alpha-linolenic acid; EPA, eicosapentaenoic acid; DPA, docosapentaenoic acid; DHA, docosahexaenoic acid.

## References

[1] Australian Bureau of Statistics Overweight and Obesity. https://www.abs.gov.au/ausstats/abs@.nsf/Lookup/by%20Subject/4364.0.55.001~2017-18~Main%20Features~Overweight%20and%20obesity~90.

[2] Blüher M. (2013). Adipose tissue dysfunction contributes to obesity related metabolic diseases. Best Pract. Res. Clin. Endocrinol. Metab..

[3] Swinburn B.A., Caterson I., Seidell J.C., James W.P.T. (2004). Diet, nutrition and the prevention of excess weight gain and obesity. Public Health Nutr..

[4] Swanson D., Block R., Mousa S.A. (2012). Omega-3 fatty acids EPA and DHA: Health benefits throughout life. Adv. Nutr. (Bethesda).

[5] Micallef M., Munro I., Phang M., Garg M. (2009). Plasma n-3 polyunsaturated fatty acids are negatively associated with obesity. Br. J. Nutr..

[6] Mingay E., Veysey M., Lucock M., Niblett S., King K., Patterson A., Garg M. (2016). Sex-dependent association between omega-3 index and body weight status in older Australians. J. Nutr. Intermed. Metab..

[7] Cazzola R., Rondanelli M., Russo-Volpe S., Ferrari E., Cestaro B. (2004). Decreased membrane fluidity and altered susceptibility to peroxidation and lipid composition in overweight and obese female erythrocytes. J. Lipid Res..

[8] Burdge G.C., Wootton S.A. (2002). Conversion of α-linolenic acid to eicosapentaenoic, docosapentaenoic and docosahexaenoic acids in young women. Br. J. Nutr..

[9] Burdge G.C., Jones A.E., Wootton S.A. (2002). Eicosapentaenoic and docosapentaenoic acids are the principal products of alpha-linolenic acid metabolism in young men. Br. J. Nutr..

[10] Jeromson S., Gallagher I., Galloway S., Hamilton D. (2015). Omega-3 Fatty Acids and Skeletal Muscle Health. Mar. Drugs.

[11] Luchtman D.W., Song C. (2013). Cognitive enhancement by omega-3 fatty acids from child-hood to old age: Findings from animal and clinical studies. Neuropharmacology.

[12] Calder P.C. (2014). Very long chain omega-3 (n-3) fatty acids and human health. Eur. J. Lipid Sci. Technol..

[13] Stillwell W., Wassall S.R. (2003). Docosahexaenoic Acid: Membrane Properties of a Unique Fatty Acid.

[14] Sun Q., Ma J., Campos H., Hankinson S.E., Hu F.B. (2007). Comparison between plasma and erythrocyte fatty acid content as biomarkers of fatty acid intake in US women.(Original Research Communications)(Author abstract). Am. J. Clin. Nutr..

[15] Lopez-Garcia E., Schulze M.B., Manson J.E., Meigs J.B., Albert C.M., Rifai N., Willett W.C., Hu F.B. (2004). Consumption of (n-3) fatty acids is related to plasma biomarkers of inflammation and endothelial activation in women. J. Nutr..

[16] Von Schacky C. (2007). n-3 PUFA in CVD: Influence of cytokine polymorphism. Proc. Nutr. Soc..

[17] Davidson M.H. (2006). Mechanisms for the Hypotriglyceridemic Effect of Marine Omega-3 Fatty Acids. Am. J. Cardiol..

[18] Li Q., Wang M., Tan L., Wang C., Ma J., Li N., Li Y., Xu G., Li J. (2005). Docosahexaenoic acid changes lipid composition and interleukin-2 receptor signaling in membrane rafts. J. Lipid Res..

[19] Leaf X.A., Kang E.J., Xiao E.Y.-F., Billman E.G. (2003). Clinical Prevention of Sudden Cardiac Death by n-3 Polyunsaturated Fatty Acids and Mechanism of Prevention of Arrhythmias by n-3 Fish Oils. Circ. J. Am. Heart Assoc..

[20] Martínez-Fernández L., Laiglesia L.M., Huerta A.E., Martínez J.A., Moreno-Aliaga M.J. (2015). Omega-3 fatty acids and adipose tissue function in obesity and metabolic syndrome. Prostaglandins Other Lipid Mediat..

[21] Munro I.A., Garg M.L. (2013). Prior supplementation with long chain omega-3 polyunsaturated fatty acids promotes weight loss in obese adults: A double-blinded randomised controlled trial. Food Funct..

[22] Innes J.K., Calder P.C. (2018). Omega-6 fatty acids and inflammation. Prostaglandins Leukot. Essent. Fat. Acids.

[23] Simopoulos A.P. (2016). An increase in the omega-6/omega-3 fatty acid ratio increases the risk for obesity. Nutrients.

[24] Australian Bureau of Statistics Fat. http://www.abs.gov.au/ausstats/abs@.nsf/Lookup/by%20Subject/4364.0.55.007~2011-12~Main%20Features~Fat~707.

[25] Jacka F.N., Pasco J.A., Williams L.J., Meyer B.J., Digger R., Berk M. (2013). Dietary intake of fish and PUFA, and clinical depressive and anxiety disorders in women. Br. J. Nutr..

[26] Meyer B. (2011). Are we consuming enough long chain omega-3 polyunsaturated fatty acids for optimal health?. Prostaglandins Leukot. Essent. Fat. Acids (Plefa).

[27] Meyer B.J. (2016). Australians are not Meeting the Recommended Intakes for Omega-3 Long Chain Polyunsaturated Fatty Acids: Results of an Analysis from the 2011–2012 National Nutrition and Physical Activity Survey. Nutrients.

[28] Ball K., Crawford D., Ireland P., Hodge A. (2003). Patterns and demographic predictors of 5-year weight change in a multi-ethnic cohort of men and women in Australia. Public Health Nutr..

[29] Mishra G.D., Loxton D., Anderson A., Hockey R., Powers J., Brown W.J., Dobson A.J., Duffy L., Graves A., Harris M. (2015). Health and Wellbeing of Women Aged 18–23 in 2013 and 1996: Findings from the Australian Longitudinal Study on Women’s Health.

[30] Sivayoganathan D., Maruthini D., Glanville J.M., Balen A.H. (2011). Full investigation of patients with polycystic ovary syndrome (PCOS) presenting to four different clinical specialties reveals significant differences and undiagnosed morbidity. Hum. Fertil..

[31] Childs C.E., Romeu-Nadal M., Burdge G.C., Calder P.C. (2008). Gender differences in the n-3 fatty acid content of tissues. Proc. Nutr. Soc..

[32] Giltay E.J., Gooren L.J., Toorians A.W., Katan M.B., Zock P.L. (2004). Docosahexaenoic acid concentrations are higher in women than in men because of estrogenic effects. Am. J. Clin. Nutr..

[33] Bakewell L., Burdge G.C., Calder P.C. (2006). Polyunsaturated fatty acid concentrations in young men and women consuming their habitual diets. Br. J. Nutr..

[34] Brown A.J., Pang E., Roberts D. (1991). Persistent changes in the fatty acid composition of erythrocyte membranes after moderate intake of n−3 polyunsaturated fatty acids: Study design implications. Am. J. Clin. Nutr..

[35] Harris W.S., Von Schacky C. (2004). The Omega-3 Index: A new risk factor for death from coronary heart disease?. Prev. Med..

[36] Katan M., Deslypere J., Van Birgelen A., Penders M., Zegwaard M. (1997). Kinetics of the incorporation of dietary fatty acids into serum cholesteryl esters, erythrocyte membranes, and adipose tissue: An 18-month controlled study. J. Lipid Res..

[37] Burrows T., Collins C., Garg M. (2011). Omega-3 index, obesity and insulin resistance in children. Int. J. Pediatric Obes..

[38] Cook R.L., O’Dwyer N.J., Donges C.E., Parker H.M., Cheng H.L., Steinbeck K.S., Cox E.P., Franklin J.L., Garg M.L., Rooney K.B. (2017). Relationship between obesity and cognitive function in young women: The food, mood and mind study. J. Obes..

[39] Young I., Parker H.M., Rangan A., Prvan T., Cook R.L., Donges C.E., Steinbeck K.S., O’Dwyer N.J., Cheng H.L., Franklin J.L. (2018). Association between haem and non-haem iron intake and serum ferritin in healthy young women. Nutrients.

[40] World Health Organisation Mean Body Mass Index (BMI). https://www.who.int/gho/ncd/risk_factors/bmi_text/en/.

[41] Delbridge E., Proietto J. (2006). State of the science: VLED (Very Low Energy Diet) for obesity. Asia Pac. J. Clin. Nutr..

[42] Schofield W.N., Schofield C., James W.P.T. (1985). Basal Metabolic Rate: Review and Prediction, Together with an Annotated Bibliography of Source Material.

[43] Morley J.J., Kushner I. (1982). SERUM C-REACTIVE PROTEIN LEVELS IN DISEASE. Ann. N. Y. Acad. Sci..

[44] Alberti K.G.M.M., Zimmet P., Shaw J. (2006). Metabolic syndrome—A new world-wide definition. A consensus statement from the international diabetes federation. Diabet. Med..

[45] Ferguson J.J., Veysey M., Lucock M., Niblett S., King K., MacDonald-Wicks L., Garg M.L. (2016). Association between omega-3 index and blood lipids in older Australians. J. Nutr. Biochem..

[46] Harris W.S. (2008). The omega-3 index as a risk factor for coronary heart disease. Am. J. Clin. Nutr..

[47] Cancer Council Victoria Dietary Questionnaires. http://www.cancervic.org.au/aboutour/research/epidemiology/nutritional_assessment_services.

[48] Ambrosini G., Mackerras D., De Klerk N., Musk A. (2003). Comparison of an Australian food-frequency questionnaire with diet records: Implications for nutrition surveillance. Public Health Nutr..

[49] Australian Department of Health The Australian Guide to Helathy Eating. http://www.health.gov.au/internet/publications/publishing.nsf/Content/nhsc-guidelines~aus-guide-healthy-eating.

[50] Willett W.C., Howe G.R., Kushi L.H. (1997). Adjustment for total energy intake in epidemiologic studies. Am. J. Clin. Nutr..

[51] Craig C.L., Marshall A.L., Sjöström M., Bauman A.E., Booth M.L., Ainsworth B.E., Pratt M., Ekelund U., Yngve A., Sallis J.F. (2003). International physical activity questionnaire: 12-country reliability and validity. Med. Sci. Sports Exerc..

[52] Australian Department of Health (2017). Recommendations to Reduce Chronic Disease Risk.

[53] Scaglioni S., Verduci E., Salvioni M., Bruzzese M.G., Radaelli G., Zetterström R., Riva E., Agostoni C. (2006). Plasma long-chain fatty acids and the degree of obesity in Italian children. Acta Paediatr..

[54] Karlsson M., Mårild S., Brandberg J., Lönn L., Friberg P., Strandvik B. (2006). Serum Phospholipid Fatty Acids, Adipose Tissue, and Metabolic Markers in Obese Adolescents*. Obesity.

[55] Gil-Campos M., Carmen Ramírez-Tortosa M., Larqué E., Linde J., Aguilera C.M., Cañete R., Gil A. (2008). Metabolic Syndrome Affects Fatty Acid Composition of Plasma Lipids in Obese Prepubertal Children. Lipids.

[56] Couet C., Delarue J., Ritz P., Antoine J., Lamisse F. (1997). Effect of dietary fish oil on body fat mass and basal fat oxidation in healthy adults. Int. J. Obes..

[57] Singla P., Bardoloi A., Parkash A.A. (2010). Metabolic effects of obesity: A review. World J. Diabetes.

[58] Alsharari Z.D., Risérus U., Leander K., Sjögren P., Carlsson A.C., Vikström M., Laguzzi F., Gigante B., Cederholm T., De Faire U. (2017). Serum fatty acids, desaturase activities and abdominal obesity–a population-based study of 60-year old men and women. PLoS ONE.

[59] Cazzola R., Rondanelli M., Trotti R., Cestaro B. (2011). Effects of weight loss on erythrocyte membrane composition and fluidity in overweight and moderately obese women. J. Nutr. Biochem..

[60] Haugaard S.B., Madsbad S., Høy C.E., Vaag A. (2006). Dietary intervention increases n-3 long-chain polyunsaturated fatty acids in skeletal muscle membrane phospholipids of obese subjects. Implications for insulin sensitivity. Clin. Endocrinol..

[61] Ghosh A., Bose K., Chaudhuri D., Baran A. (2003). Association of food patterns, central obesity measures and metabolic risk factors for coronary heart disease (CHD) in middle aged Bengalee Hindu men, Calcutta, India. Asia Pac. J. Clin. Nutr..

[62] Bray G.A., Clearfield M.B., Fintel D.J., Nelinson D.S. (2009). Overweight and obesity: The pathogenesis of cardiometabolic risk. Clin. Cornerstone.

[63] Parra D., Ramel A., Bandarra N., Kiely M., Martínez J.A., Thorsdottir I. (2008). A diet rich in long chain omega-3 fatty acids modulates satiety in overweight and obese volunteers during weight loss. Appetite.

[64] Udani J.K., Ritz B.W. (2013). High potency fish oil supplement improves omega-3 fatty acid status in healthy adults: An open-label study using a web-based, virtual platform. Nutr. J..

[65] Hibbeln J.R. (2002). Seafood consumption, the DHA content of mothers’ milk and prevalence rates of postpartum depression: A cross-national, ecological analysis. J. Affect. Disord..

[66] Holman R.T., Johnson S.B., Ogburn P.L. (1991). Deficiency of essential fatty acids and membrane fluidity during pregnancy and lactation. Proc. Natl. Acad. Sci. USA.

[67] Kristal A.R., Peters U., Potter J.D. (2005). Is it time to abandon the food frequency questionnaire?. Cancer Epidemiol. Biomark. Prev..

[68] Andersen L.F., Tomten H., Haggarty P., Løvø A., Hustvedt B. (2003). Validation of energy intake estimated from a food frequency questionnaire: A doubly labelled water study. Eur. J. Clin. Nutr..

[69] Kaskoun M.C., Johnson R.K., Goran M.I. (1994). Comparison of energy intake by semiquantitative food-frequency questionnaire with total energy expenditure by the doubly labeled water method in young children. Am. J. Clin. Nutr..

[70] Sam S., Dunaif A. (2003). Polycystic ovary syndrome: Syndrome XX?. Trends Endocrinol. Metab..

